# Molecular characterization of extended-spectrum beta-lactamase-producing bacteria isolated from pregnant women’s urine at Itojo Hospital, South Western Uganda

**DOI:** 10.1099/acmi.0.001045.v5

**Published:** 2026-03-11

**Authors:** Muzafaru Twinomujuni, Benson Musinguzi, Moses Asiimwe, Stephen Samuel Mpiima, Henry Zamarano, Isaac Orikushaba, Deus Muhanguzi, Crinad Twinamatsiko, Sarapia Paul Mallya, Jamiru Samiri, Joseph Kamugisha, Pauline Petra Nalumaga, Taseera Kabanda, Kennedy Kassaza, Charles Nkubi Bagenda, Barbra Tuhamize, Joel Bazira, Rosemary Ricciardelli, Moses Mpeirwe

**Affiliations:** 1 Department of Microbiology and Parasitology, Faculty of Medicine, Mbarara University of Science and Technology, Mbarara City P.O. Box 1410, Uganda; 2 Department of Medical Laboratory Sciences, Faculty of Health Sciences, Muni University, P.O. Box, 725 Arua City, Uganda; 3 Department of Immunology and Molecular Biology, School of Biomedical Sciences, College of Health Sciences, Makerere University, Kampala P.O Box 7072, Uganda; 4 Department of Medical Laboratory Science, Faculty of Medicine, Mbarara University of Science and Technology, P.O. Box 1410 Mbarara City, Uganda.; 5 Memorial University of Newfoundland, 155 Ridge Road, St. John’s A1C 5R3, NL, Canada

**Keywords:** antimicrobial, antenatal clinic, extended spectrum *β*-lactamase, pregnancy

## Abstract

**Background.** Extended-spectrum *β*-lactamase (ESBL)-producing bacteria pose a global challenge because of resistance developing against a wide range of antimicrobial agents, complicating available treatment options. Thus, identifying the prevalent bacterial species producing ESBL enzymes and understanding how they are susceptible to antibiotics is necessary to inform effective treatment guidelines.

**Objective.** We sought to characterize ESBL-producing bacteria isolated from pregnant women’s urine at Itojo Hospital, Ntungamo district, Southwestern Uganda.

**Methods.** We conducted a cross-sectional study where we collected and analysed 340 urine samples from 340 pregnant women. We did antimicrobial susceptibility testing using the Kirby–Bauer disc diffusion method. Isolates were screened for ESBL production and confirmed using the combination disc test. Genotypic characterization was confirmed using multiplex PCR to detect *bla*TEM, *bla*CTX-M and *bla*SHV genes.

**Results.** The prevalence of ESBL-producing bacteria was 29.7% (101/340). *Escherichia coli* 36/101 (35.6%) and *Klebsiella* species 33/101 (32.7%) were predominant ESBL producers. Genotypic analysis revealed *bla*TEM 50/101 (49.5%) and *bla*CTX-M 31/101 (30.7%) as the most prevalent genes, while *bla*SHV was less common, 8/101 (7.9%)

**Conclusion.** The high prevalence of ESBL-producing bacteria and their resistance to commonly used antibiotics highlighted the need for targeted antibiotic therapy, antimicrobial stewardship and regular molecular surveillance.

## Data Summary

All data generated or analysed during this study are included in this research article.

## Introduction

Urinary tract infections (UTIs) are among the most common bacterial infections during pregnancy, accounting for ~25% of all infections in pregnant women [1,2]. The hormonal and physiological changes that occur during pregnancy are known to increase women’s susceptibility to UTIs, which can lead to complications if left untreated [3,4]. Thus, more research on antimicrobial resistance is needed to better support pregnant women. In addition, beyond UTIs being uncomfortable (even painful), in part due to the close positioning of the urinary tract to the uterus, complications are possible for the mother and fetus. Receiving timely, accurate diagnosis and adequate therapy reduces the risk of developing problems from medication resistance to UTIs [5], necessary, given that UTIs and their related complications are sadly responsible for ~150 million fatalities in these populations annually [6].

In clinical and community settings, bacterial infections have increased dramatically because, for instance, Gram-negative bacteria like *Klebsiella pneumoniae* and *Escherichia coli* develop extended-spectrum beta-lactamases (ESBL) enzymes that hydrolyse beta-lactam rings of several antibiotics [7]. Simply said, there is resistance to therapies.

In part for these reasons, scholars do pay more attention to the clinical context in the fight against antibiotic resistance, which constitutes an international challenge [8,10]. However, the severity of the danger of UTIs in community settings remains unknown and understudied. The limited scholarship available suggests, as noted by Frost *et al.* [11], that ESBL-producing *Enterobacteriaceae* are a growing international concern for UTIs due to their resistance to beta-lactam antibiotics, including penicillin and third-generation cephalosporin. These organisms account for an estimated 25–50% of UTIs worldwide, with 20–40% occurring in healthy community populations. The emergence of ESBL-producing *Enterobacteriaceae* in UTIs presents a substantial public health concern, particularly in hospital settings [12]. The emergence of resistance in ESBL-producing bacteria in UTIs is further intensified by the excessive or improper use of antibiotics, particularly cephalosporins and monobactams, which are hydrolysed by ESBLs. The dispersion of the drug-resistant bacteria makes managing infections caused by ESBL-producing strains increasingly difficult [13].

ESBL-producing bacteria threaten public health, particularly in healthcare settings. In response, our study aimed to investigate the high prevalence of ESBL-resistant bacteria in UTIs among women who are pregnant. Specifically, we characterized the specific ESBL genes circulating in the Itojo Hospital region, a major health facility supporting other facilities where there was an observed increase in pregnant women presenting with UTIs. For example, in 2022, the prevalence of UTIs among antenatal attendees was 15% or ~792 out of 5,280 pregnant women. Concerningly, by 2024, the figure had increased significantly to 29%, with 1,531 of 5,280 pregnant women diagnosed with UTIs. We characterized ESBL-producing bacteria isolated from pregnant women’s urine at Itojo Hospital, Ntungamo District, Southwestern Uganda.

## Methods

The sample size for the participants was estimated using Krejcie and Morgan (14). The calculation was based on an estimated population proportion of 0.50, with a margin of error of 0.05 and chi-square values for 1 degree of freedom at the specified confidence interval of 3.843. The sample size was therefore estimated to be 340 participants. We surveyed pregnant women who attended the antenatal clinic at Itojo Hospital and voluntarily consented to participate in the study.

### Inclusion and exclusion criteria

Inclusion required participants to be pregnant women, presented with or without symptoms or clinical signs suggestive of a urinary tract infection. As such, we excluded pregnant and non-pregnant women with allergies to antibiotics, a history of renal impairment or other chronic conditions, and a history of antibiotic use within 2 weeks prior to their presentation at the antenatal clinic. Patients with renal impairment were excluded to ensure the study’s findings were not confounded by pre-existing conditions that can complicate UTIs and influence bacterial profiles. Their immune response and drug metabolism can be different, which might affect the type of bacteria present and their susceptibility to antibiotics, thus potentially skewing the study’s results on the general population. Patients who had used antibiotics within 2 weeks before the study were excluded to ensure the accuracy of the culture results. When a patient is on antibiotics, the medication can suppress or eliminate the bacteria in their urine. This can lead to a false-negative culture result, where an infection is present but the lab test shows no bacterial growth. By excluding these patients, we were able to isolate and characterize bacteria from true, active infections, providing a more accurate representation of ESBL prevalence in our target population. This approach allows for a more reliable assessment of antibiotic resistance patterns.

### Procedure

We registered study participants with a participation identification number, rather than using their names, for anonymity and confidentiality, while cross-checking for accurate data entry and completeness. We consented participants, confirmed confidentiality and double-checked their demographic information.

### Urine sample collection, handling and bacterial isolation and identification

Participants took a mid-stream urine sample using a labelled, sterile, dry, wide-necked, leak-proof urine container measuring 10 to 15 ml. We collected 340 urine samples from 340 pregnant women. Using a calibrated urine loop (1/1,000 ml), we inoculated the urine samples on Cystine Lactose Electrolyte Deficient Agar (CLED) (Oxoid Ltd, UK) medium and cultured aerobically at 37 °C for 18 to 24 h (i.e. overnight culture). We checked the plates for growth after an overnight incubation, and the degree of growth was determined by colony count. Bacterial colony counts of at least 10^5^ c.f.u. ml^−1^ were significant, and counts between 10^2^ and 10^4^ c.f.u. ml^−1^ were doubtful, and colony counts below 10^2^ c.f.u. ml^−1^ were insignificant. Additional identification tests were performed on cultures showing significant bacteriuria. Separate colonies on CLED agar plates that showed significant growth were selected, inoculated on MacConkey agar and Blood agar and incubated for 18 to 24 h at 37 °C to differentiate *Enterobacteriaceae*. We identified the organisms from the pure culture plate by the Gram staining method and other biochemical tests. We identified enterobacteria, like *Proteus* spp., *E. coli* and distinguished *Proteus vulgaris* from *Proteus mirabilis* using an indole test. We also used an oxidase test to identify non-lactose fermenting bacteria, mainly *Pseudomonas* spp., which we distinguished from other enterobacteria by a urease test. The distinct enterobacteria were distinguished by the triple sugar iron agar test. All subsequent biochemical analyses were performed according to accepted clinical laboratory practices.

### Antimicrobial susceptibility testing

We used the Clinical and Laboratory Standards Institute (CLSI) Kirby-Bauer disc diffusion method for antimicrobial susceptibility testing. The study obtained bacterial inocula comparable to 0.5 McFarland turbidity standards by suspending pure culture colonies of 24 h growth in a tube with 4 ml of sterile physiological saline.

### Combined disk test

We used a marked difference in the zone widths of inhibition between the ceftazidime–clavulanic acid disc and the ceftazidime-alone disc to indicate the presence of ESBL formation. We used CLSI 2024 guidelines, which stated that a difference of ≥5 mm in the diameter of the inhibition zone between the antibiotic alone and the antibiotic plus clavulanic acid is considered significant and indicative of ESBL production. The diameter of the inhibition zone was measured using a ruler and recorded in millimetres. Measurement was taken from edge to edge across the centre of the antibiotic disc, including the disc itself, to ensure accurate and consistent results. We detected ESBL-producing bacteria based on differences in zone sizes when a predetermined cut-off point was exceeded.

### Genotypic screening of ESBL

Using the Gene JET Genomic DNA Purification Kit from Thermo Scientific, we isolated crude genomic bacterial DNA from all isolates exhibiting the positive ESBL phenotype. We used a traditional, multiplex PCR experiment using a PCR master mix (DreamTaq Green PCR Master Mix; Thermo Scientific) and specific primers to screen for ESBL genes: *bla*TEM, *bla*SHV and *bla*CTX-M.

### Primer design and selection

Each of the ORFs of specific targets was amplified using forward and reverse primers, each with a unique sequence, concentration and annealing temperature (see Table 1).

**Table 1.** Nucleotide sequence of PCR primers used to amplify the ESBL encoding genes

Primer	Sequence 5^1^–**3^1^**	Conc (µm)	Amplicon size (bp)	Annealing temp (°C)
CTX-M	F-CGCTTTGCGATGTGCAG R-ACCGCGATATCGTTGGT	10	550	51.5
TEM	F-CATTTCCGTGTCGCCCTTATTC R-CGTTCATCCATAGTTGCCTGAC	15	800	51.5
SHV	F-GGTTATGCGTTAATTCGCC R-TTAGCGTTGCCAGTGCTC	10		51.5

### PCR amplification/cycling

We completed the PCR amplification using a conventional PCR Thermocycler (CLASSIC K960 Thermal Cycler). The programme included initial denaturation at 95 °C for 3 min, followed by 40 cycles of denaturation at 95 °C for 30 s, annealing at 51.2 °C for 30 s and elongation at 72 °C for 1 min, before the final extension cycle of 72 °C for 5 min.

### Gel electrophoresis

**Fig. 1.**
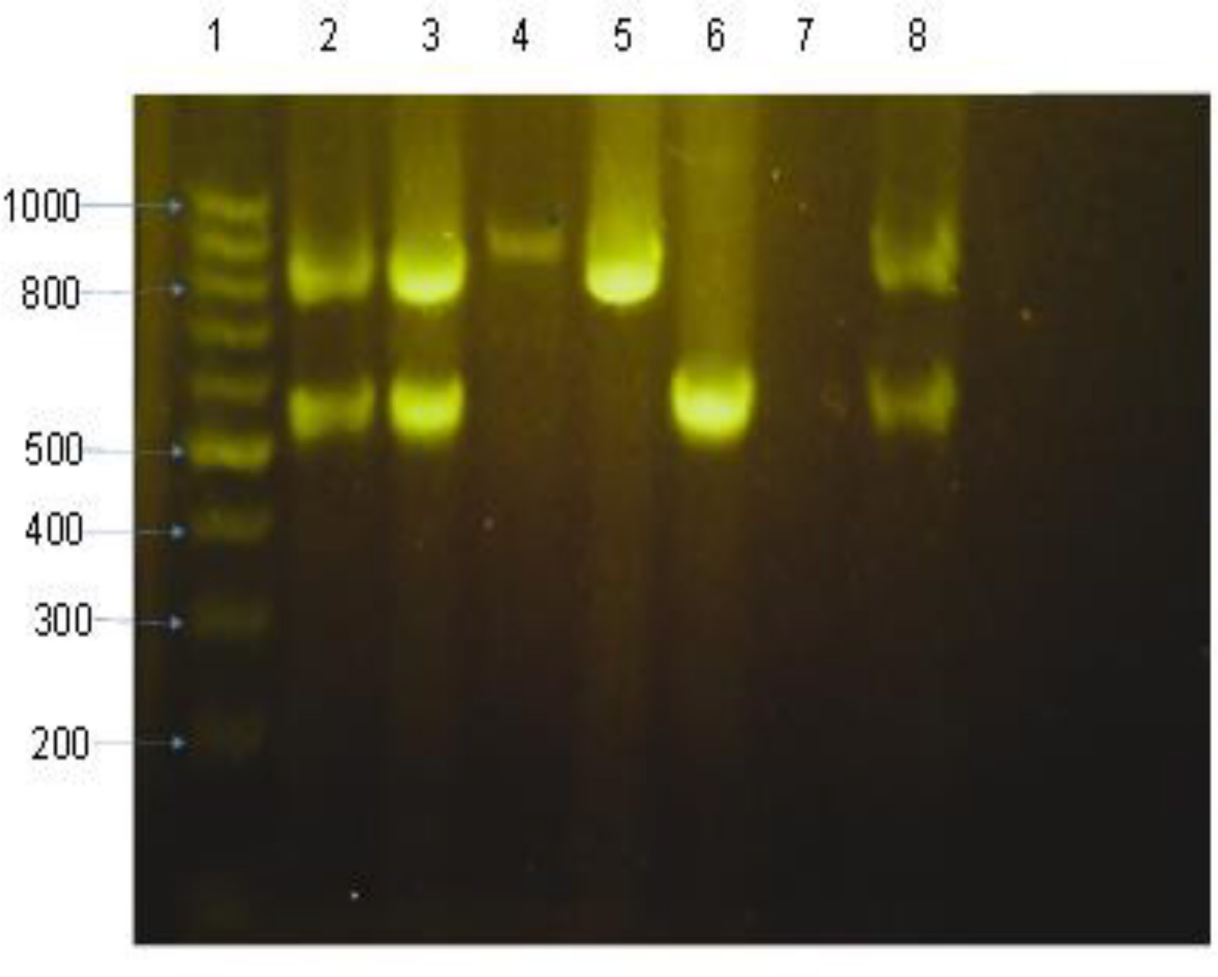
Agarose gel electrophoresis of PCR products: 1, ladder (base pairs); 2 and 8, positive controls for duplex of TEM and CTX; 7, negative control; 3, sample containing TEM and CTX; 4, SHV alone; 5, TEM alone; 6, CTX alone.

DNA amplicon was electrophoresed using 1.5% agarose gel, in 1× Tris-Borate EDTA buffer, 5 µl DSView^™^ Nucleic acid stain (cat. no.: M7011), 6X loading buffer (GDSBio Lot 050) and DNA ladder/marker 100 bp (GDSBio Lot 076). Electrophoresis was run at 200 V and 80 mA for 1 h. We visualized bands using the Gene-Flash Trans-illuminator, as shown in Fig. 1.

### Data quality control

We evaluated and verified all of the data collected by having the lead researcher ensure all pre-analytical, analytical and post-analytical procedures were standardized and adhered to the established standard operating procedures. To detect ESBL, we did physical and chemical parameters testing, medium pH testing, sterility testing, performance and used the quality control strains of *E. coli* (ATCC^®^ 25922TM), *K. pneumoniae* ATCC 700603 (an ESBL producer) and *P. mirabilis* ATCC BAA-856 for antibiotic susceptibility testing.

### Statistical and data analysis

We entered the raw data into a Microsoft Excel spreadsheet and later imported the dataset into STATA (version 17) for statistical analysis. We computed the descriptive statistics (i.e. frequencies and percentages) of mean, median and range to describe participant characteristics; then, we used the diameter of the zones of inhibition (mm) to report the antimicrobial activity based on the CLSI 2024.

## Results

### Study participants

In total, we recruited 340 participants, from whom we collected 340 urine samples, which we tested for ESBL-producing bacteria. Out of the 340 samples collected, 101/340 (29.7%) yielded ESBL-producing bacteria positive culture and sensitivity results. The median age of the study participants was 24 years with an interquartile range of 20–27 years. Only 27.9% (*n*=95) of participants attended secondary school as their highest level of education, and (*n*=183, 53.8%) were self-employed. Here, 101 study participants had ESBL-producing bacteria; thus, the overall prevalence of such was 29.7% (see Table 2).

**Table 2.** Demographic characteristics of study participants

Variable	Total *N*=**340**	ESBL organism	
		Absent *n*=239 (70.3%)	Present *n*=101 (29.7%)
Age: median (IQR)	**24 (20–27)**	**24 (20–27)**	**25 (20–28)**
**Education**			
Secondary	95 (27.9%)	70 (74%)	25 (26%)
University	12 (3.5%)	8 (67%)	4 (33%)
None	76 (22.4%)	51 (67%)	25 (33%)
Primary	93 (27.4%)	65 (70%)	28 (30%)
Tertiary	64(18.8%)	45 (70%)	19 (30%)
**Occupation**			
Housewife	12 (3.5%)	9 (75.0%)	3 (25.0%)
Civil servant	31 (9.1%)	20 (64.5%)	11 (35.5%)
Peasant	114 (33.5%)	75 (65.8%)	39 (34.2%)
Self-employed	183 (53.8%)	135 (73.8%)	48 (26.2%)
**Parity**			
0	53 (15.6%)	37 (69.8%)	16 (30.2%)
1–4	268 (78.8%)	191 (71.3%)	77 (28.7%)
≥5	19 (5.6%)	11 (57.9%)	8 (42.1%)

### The prevalence of ESBL-producing bacteria

**Fig. 2.**
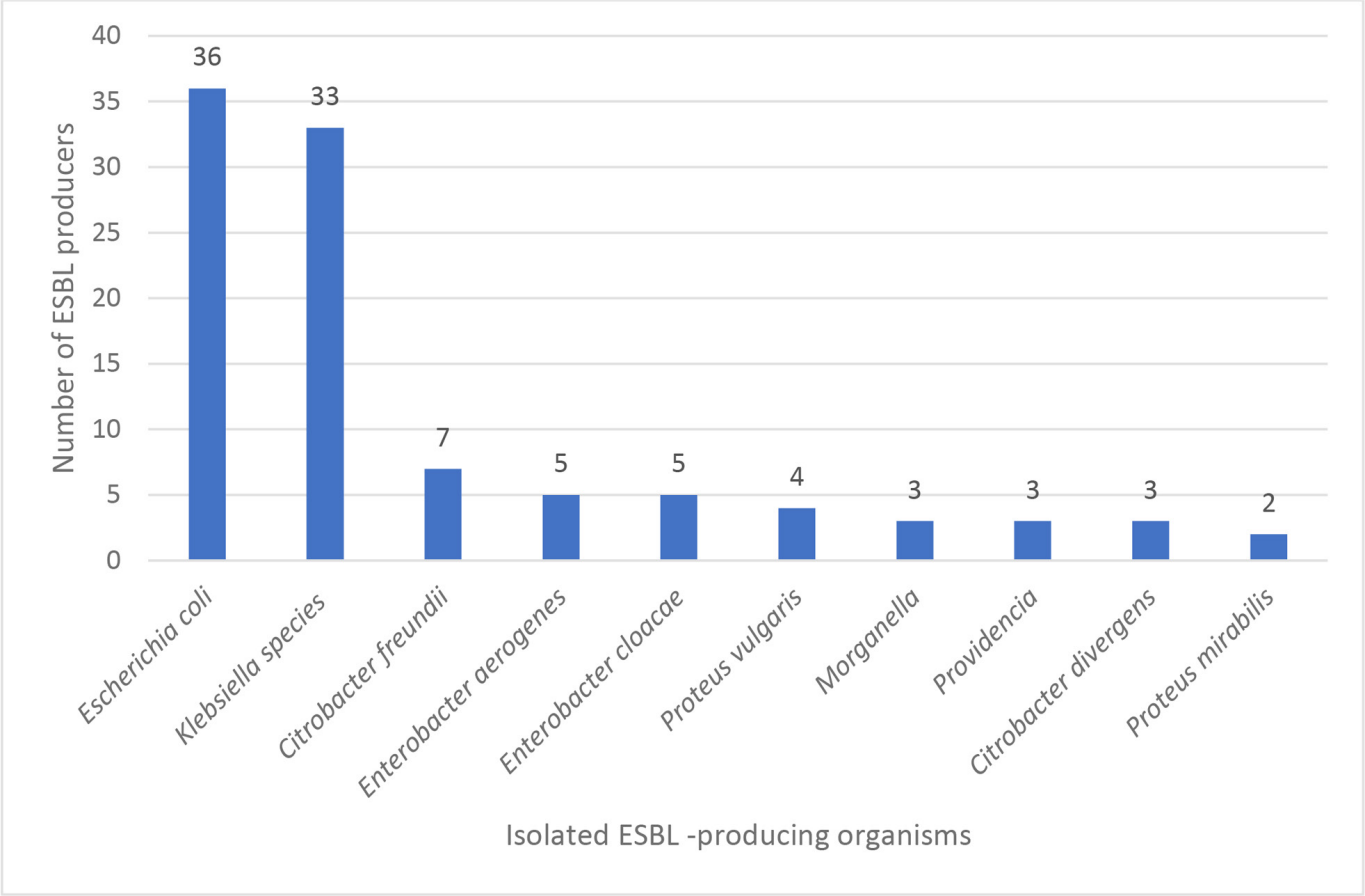
Prevalence of ESBL-producing bacteria.

ESBL-producing organisms were identified phenotypically using the combined disc test. Among these, the majority were * E. coli* 36/101 (35.64%), followed by *Klebsiella* species 33/101 (32.67%), *Citrobacter freundii* 7/101 (6.93%), *Enterobacter aerogenes* 5/101 (4.95%), *Enterobacter cloacae* 5/101 (4.95%), *P. vulgaris* 4/101 (3.96%), *Citrobacter divergens* 3/101 (2.97%), *Morganella* species 3/1,013/101 (2.97%) and *Providencia* species 3/101 (2.97%), and the minimal frequent ESBL-producing organism was *P. mirabilis* 2/101 (1.98%) (see Fig. 2).

### The antimicrobial susceptibility profile of ESBL-producing organisms

The isolated ESBL-producing organisms were identified according to the Clinical and Laboratory Standards Institute 2024. We used the antibiotic discs (Oxoid, England) of cefotaxime (CTX) (5 µg), cefotaxime/clavulanic acid (CEC) (30/10 µg), ceftazidime (CAZ) (30 µg), ceftazidime/clavulanic acid (CAC) (30/10 µg), imipenem (IPM) (10 µg), nitrofurantoin (F) (10 µg), ciprofloxacin (CIP) (5 µg) and ceftriaxone (CRO) (30 µg) (see Table 3).

**Table 3.** Antimicrobial susceptibility profile of the ESBL-producing organisms

Isolated organism	Antibiotic discs used																							
	CTX			CEC			CAZ			CAC			IPM			F			CIP			CRO		
	S	I	R	S	I	R	S	I	R	S	I	R	S	I	R	S	I	R	S	I	R	S	I	R
*Escherichia coli* (36)	9	0	27	27	0	9	19	0	17	17	0	9	24	0	12	12	0	25	25	0	11	11	0	25
*Klebsiella* species (33)	8	0	25	25	0	8	19	0	14	14	0	9	24	0	9	9	0	22	22	0	11	11	0	22
*C. freundii* (7)	0	0	7	7	0	0	3	0	4	4	0	3	4	0	3	3	0	4	4	0	3	3	0	4
*C. divergens* (3)	1	0	2	2	0	1	2	0	1	1	0	2	2	0	1	1	0	2	2	0	1	1	0	2
*Enterobacter aerogenes* (5)	1	0	4	4	0	1	2	0	2	3	0	2	2	0	3	3	0	2	3	0	2	2	0	3
*Enterobacter cloacae* (5)	3	0	2	2	0	3	4	0	1	1	0	4	5	0	0	0	0	5	3	0	2	2	0	3
*P. vulgaris* (4)	2	0	2	2	0	2	3	0	1	1	0	3	3	0	1	13	0	3	2	0	2	2	0	2
*Morganella* (3)	1	0	2	2	0	1	3	0	0	0	0	3	2	0	1	1	0	2	2	0	1	1	0	2
*Providencia* (3)	1	0	2	2	0	1	0	0	3	3	0	0	1	0	2	2	0	1	2	0	1	1	0	2
*P. mirabilis* (2)	1	0	1	1	0	1	1	0	1	1	0	1	1	0	1	1	0	1	1	0	1	1	0	1

S, sensitive; I, intermediate; R, resistant. No isolate had an intermediate antimicrobial profile.

### Genotypic characterization of ESBL-producing organisms

**Fig. 3.**
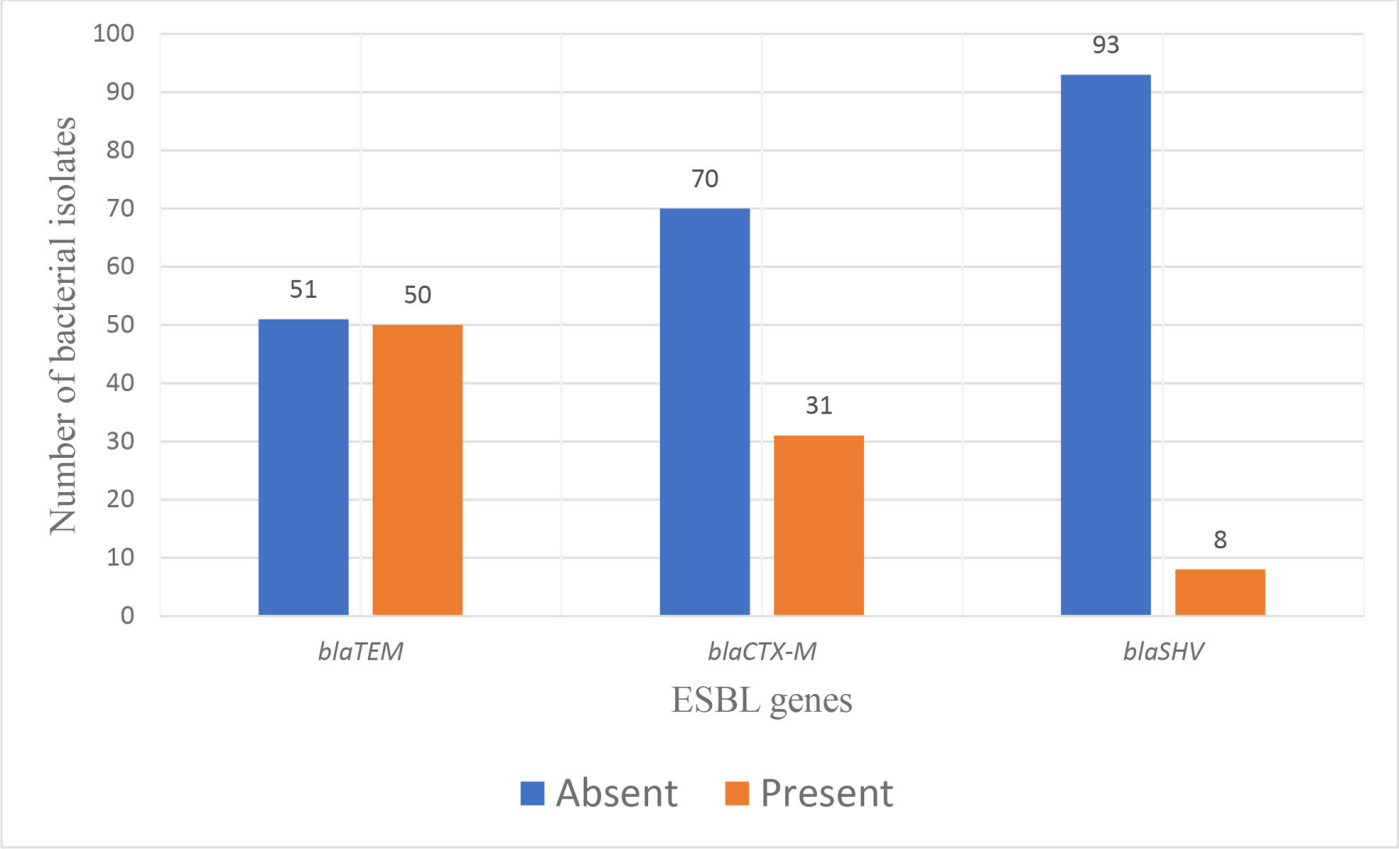
Prevalence of common ESBL genes among bacterial isolates. This bar graph illustrates the number of bacterial isolates with either presence or absence of the three key ESBL-encoding genes: *bla*TEM, *bla*CTX-M and *bla*SHV. The data show that the *bla*SHV gene was the least common, found in 8 isolates, while *bla*TEM and *bla*CTX-M were detected in 50 and 31 isolates, respectively.

We completed the genotypic analysis of all ESBL-producing bacterial isolates. The *bla*TEM encoding genes were 49.5% (50/101) present and 50.5% (*n*=51/101) absent for ESBL-encoding or non-ESBL-encoding, respectively. The comparable figures suggested that *bla*TEM genes are essential for the genotypic analysis of ESBL generation. The *bla*CTX-M encoding genes were 69.3% (70/101) absent *bla*CTX-M and 30.7% (31/101) present, indicating a significant proportion of *bla*CTX-M genes do encode for ESBLs. Most of the *bla*SHV genes, 92.1% (93/101), were absent. Thus, they did not encode for ESBLs. In contrast, only 7.9% (8/101) of *bla*SHV genes were present, indicating *bla*SHV genes are less commonly associated with ESBL production (see Fig. 3).

### The distribution of isolated organisms against ESBL-encoding genes

*E. coli* and *Klebsiella* species were the most prevalent ESBL producers; they frequently tested negative for all the specific encoding genes analysed in this study.

The highest number of isolates showing genotype–absent but phenotype–presence results was observed across all three ESBL-encoding genes. This indicated the presence of phenotypic resistance to extended-spectrum beta-lactams despite the absence of detectable *bla*TEM, *bla*CTX-M or *bla*SHV genes, suggesting the possible involvement of other resistance mechanisms or gene variants not targeted in the current study (see Table 4).

**Table 4.** Distribution of isolated organisms against ESBL-encoding genes

Organism	Total	*bla*SHV absent	*bla*SHV present	*bla*CTX-M present	*bla*CTX-M absent	*bla*TEM absent	*bla*TEM present
*C. divergens*	3	0	3	2	1	2	1
*C. freundii*	7	0	7	5	2	5	2
*Enterobacter aerogenes*	5	0	5	1	4	5	0
*Enterobacter cloacae*	5	2	3	0	5	4	1
*Escherichia coli*	36	1	35	14	22	12	24
*Klebsiella* species	33	4	29	8	25	20	13
*Morganella*	3	0	3	0	3	3	0
*P. mirabilis*	2	1	1	1	1	1	1
*P. vulgaris*	4	0	4	0	4	1	3
*Providencia*	3	0	3	0	3	2	1
**Total**	101	8	93	31	70	51	50

## Discussion

In the current study, we aimed to characterize bacteria isolated from pregnant women’s urine at Itojo Hospital in Ntungamo District, Southwestern Uganda. The overall prevalence of ESBL-producing bacteria was 29.7%, aligning closely with findings from Minia, Egypt, where a 32.5% prevalence was reported [14].

In our study, *E. coli* (35.64%) and *Klebsiella* species (32.67%) were the most common ESBL producers, aligning with findings by Acaku *et al.* [15] and Verma *et al.* [16]. Acaku reported *E. coli at* 42.9% and *K. pneumoniae* at 28.6%, while Verma found * E. coli* at 47.5% and *Klebsiella* spp. at 34.1%. These studies consistently identified the same predominant species, with variations in their relative proportions.

Antimicrobial susceptibility testing revealed high levels of resistance among ESBL-producing isolates. *E. coli* (75%), *Klebsiella* spp. (75.8%) and *C. freundii* (100%*)* exhibited marked resistance to cefotaxime. Similarly, notable resistance to cefotaxime/clavulanic acid was observed in *E. coli* (42.7%), *Klebsiella* spp. (42.4%) and *C. freundii* (57.1%). Most isolates showed resistance to ciprofloxacin and ceftriaxone, mirroring reports by Jena *et al.* [17] and Mayanja *et al.* [5], who recorded resistance rates of 50% and 59% for ceftriaxone and ciprofloxacin, respectively. These findings underscored the widespread antimicrobial resistance among ESBL-producing organisms and suggested a potential link between ESBL prevalence and multidrug resistance [18].

Given the high burden of antimicrobial resistance, regular screening for ESBL-producing organisms in pregnant women is essential, particularly in antenatal clinics. Early detection helps to guide appropriate treatment, prevent complications such as pyelonephritis and preterm labour and reduce the risk of vertical transmission to neonates. Early screening facilitates infection control and informs antimicrobial stewardship strategies within healthcare facilities.

The molecular characterization revealed the presence of *bla*SHV and *bla*TEM genes, with *bla*TEM being more predominant, consistent with prior studies [17,19,21]. Despite phenotypic resistance to third-generation cephalosporins, a significant number of isolates lacked detectable ESBL-encoding genes. The discrepancy between genotype and phenotype suggested the possible involvement of other resistance mechanisms, such as plasmid-mediated AmpC *β*-lactamases, gene variants not targeted in this study or regulatory mutations influencing gene expression.

Although genotypic testing offers insights into the molecular epidemiology and potential transmission dynamics of resistance genes, our findings suggest genotypic markers alone may not reliably predict ESBL phenotypes. Therefore, a combined approach of phenotypic and genotypic testing remains crucial, especially in clinical decision-making and when investigating outbreaks.

Our results highlighted a need for tailored antibiotic therapy, routine antimicrobial susceptibility testing and implementation of antimicrobial stewardship programmes [22].

A comparison of phenotypic and genotypic results revealed how, although all 101 isolates were phenotypically confirmed as ESBL producers, only 49.5%, 30.7% and 7.9% were positive for *bla*TEM, *bla*CTX-M and *bla*SHV genes, respectively. This indicates a mismatch between phenotypic and genotypic detection, suggesting the possible presence of other ESBL genes not tested. Co-carriage of ESBL genes was observed in a few isolates, particularly among *E. coli* and *Klebsiella* species. These findings highlight the need for broader molecular screening to accurately characterize ESBL-producing bacteria [19].

We also found molecular genotyping of the ESBL-positive isolates revealed *bla*TEM as the most common gene, followed by *bla*CTX-M. In contrast, *bla*SHV was the least detected and, therefore, less commonly associated with ESBL production [17,19,21].

## Limitations

Our cross-sectional study design limited our ability to draw causal conclusions on the maternal and foetal complications related to ESBL-producing organisms. As such, longitudinal inquiry is a necessary next step.

## Conclusion

*E. coli* (35.64%) and *Klebsiella* species (32.67%) were the most common ESBL producers, highlighting these as the primary pathogens responsible for UTIs in pregnant mothers. In addition, both exhibited considerable resistance to commonly used antibiotics, such as cefotaxime, nitrofurantoin, ciprofloxacin and ceftriaxone. The organisms were more susceptible to the combination antibiotic discs, indicating that combination therapies may be more effective for treating infections caused by these resistant strains.

*bla*TEM encoding genes were found to be equally distributed between positive and negative, with *E. coli* showing the highest positivity. *bla*CTX-M genes played a significant role in ESBL production, particularly in *E. coli* and *Klebsiella* species. The *bla*SHV genes were less commonly associated with ESBL production.

The presence of multiple ESBL-encoding genes within a single organism, especially in *E. coli* and *Klebsiella* species, underlines the complexity and potential for multidrug resistance in these bacteria.

The high antibiotic drug resistance poses a concerning challenge for the management of ESBL infections, which appears likely to compel clinicians to increase their usage of antibiotics. *bla*CTX-M and *bla*TEM genes had a higher proportion of ESBL-positive cases, making them more likely contributors to antibiotic resistance through ESBL production. The majority of *blaS*HV genes were less commonly associated with ESBL production.

## Consideration

From our findings, we put forth the following considerations for practice and policy. First, given that broad-spectrum antibiotics are largely ineffective against many ESBL-producing bacteria, hospital management should implement strict antibiotic prescribing guidelines to minimize their unnecessary use. Second, regular screening and monitoring of pregnant women presenting with symptomatic UTIs for ESBL-producing bacteria is critical for early detection and initiation of appropriate treatment, thereby minimizing the risk of adverse maternal and foetal outcomes. The practice can contribute to improved antibiotic stewardship by informing targeted therapy and reducing the inappropriate use of broad-spectrum antibiotics. Furthermore, the practice aids in limiting the transmission of resistant strains within healthcare settings and facilitates the evaluation of treatment efficacy and identification of recurrent infections. Third, sensitization and awareness campaigns should be conducted for both community members and healthcare workers, focusing on the risks of drug-resistant infections, the importance of completing prescribed antibiotic courses and measures to prevent reinfection. Fourth, the use of combination antibiotic therapy, such as cefotaxime/clavulanic acid or ceftazidime/clavulanic acid, which showed greater effectiveness against the isolated organisms, should be considered for inclusion in treatment guidelines for UTIs among pregnant women. Finally, further research should be undertaken to assess the impact of ESBL-producing infections on maternal and neonatal outcomes, including longitudinal studies to explore resistance development, environmental factors and the potential of alternative therapies or natural compounds in managing these infections.

## Abbreviations

CAC: ceftazidime/clavulanic acid
CAZ: ceftazidime
CEC: cefotaxime/clavulanic acid
CIP: ciprofloxacin
CLED: Cystine Lactose Electrolyte Deficient Agar
CLSI: Clinical and Laboratory Standards Institute
CRO: ceftriaxone
CTX: cefotaxime
ESBL: extended-spectrum *β*-lactamase
F: nitrofurantoin
IPM: imipenem
UTIs: urinary tract infections
